# A characterization of Chover-type law of iterated logarithm

**DOI:** 10.1186/2193-1801-3-386

**Published:** 2014-07-28

**Authors:** Deli Li, Pingyan Chen

**Affiliations:** Department of Mathematical Sciences, Lakehead University, Thunder Bay, P7B 5E1 Ontario Canada; Department of Mathematics, Jinan University, Guangzhou, 510630 China

**Keywords:** (*α*,*β*)-Chover-type law of the iterated logarithm, Sums of i.i.d. random variables, Symmetric stable distribution with exponent *α*

## Abstract

**Abstract:**

Let 0 < *α* ≤ 2 and − *∞* <*β* <*∞*. Let {*X*_*n*_;*n* ≥ 1} be a sequence of independent copies of a real-valued random variable *X* and set *S*_*n*_ = *X*_1_+⋯+*X*_*n*_, *n* ≥ 1. We say *X* satisfies the (*α*,*β*)-Chover-type law of the iterated logarithm (and write *X*∈*CTLIL*(*α*,*β*)) if  almost surely. This paper is devoted to a characterization of *X* ∈*CTLIL*(*α*,*β*). We obtain sets of necessary and sufficient conditions for *X*∈*CTLIL*(*α*,*β*) for the five cases: *α* = 2 and 0 < *β* <*∞*, *α* = 2 and *β* = 0, 1<*α*<2 and −*∞*<*β*<*∞*, *α* = 1 and −*∞* <*β* <*∞*, and 0 < *α* <1 and −*∞* <*β* <*∞*. As for the case where *α* = 2 and −*∞* <*β* <0, it is shown that *X*∉*CTLIL*(2,*β*) for any real-valued random variable *X*. As a special case of our results, a simple and precise characterization of the classical Chover law of the iterated logarithm (i.e., *X*∈*CTLIL*(*α*,1/*α*)) is given; that is, *X*∈*CTLIL*(*α*,1/*α*) if and only if  where  whenever 1< *α* ≤ 2.

**Mathematics Subject Classification (2000):**

Primary: 60F15; Secondary: 60G50

## 1 Introduction

Throughout, {*X*_*n*_; *n* ≥ 1} is a sequence of independent copies of a real-valued random variable *X*. As usual, the partial sums of independent identically distributed (i.i.d.) random variables *X*_*n*_, *n* ≥ 1 will be denoted by . Write *Lx*= log(*e*∨*x*), *x* ≥ 0.

When *X* has a symmetric stable distribution with exponent *α*∈(0,2), i.e.,  for *t*∈(−*∞*,*∞*), Chover 1966 proved that
1.1

This is what we call the classical Chover law of iterated logarithm (LIL). Since then, several papers have been devoted to develop the classical Chover LIL. See, for example, Hedye 1969 showed that (1.1) holds when *X* is in the domain of normal attraction of a nonnormal stable law, Pakshirajan and Vasudeva 1977 discussed the limit points of the sequence , Kuelbs and Kurtz 1974 obtained the classical Chover LIL in a Hilbert space setting, Chen 2002 obtained the classical Chover LIL for the weighed sums, Vasudeva 1984, Qi and Cheng 1996, Peng and Qi 2003 established the Chover LIL when *X* is in the domain of attraction of a nonnormal stable law, Scheffler 2000 studied the classical Chover LIL when *X* is in the generalized domain of operator semistable attraction of some nonnormal law, Chen and Hu 2012extended the results of Kuelbs and Kurtz 1974 to an arbitrary real separable Banach space, and so on. It should be pointed out that the previous papers only gave sufficient conditions for the classical Chover LIL.

Motivated by the previous study of the classical Chover LIL, we introduce a general Chover-type LIL as follows.

### **Definition1.1**

Let 0 < *α* ≤ 2 and −*∞* <*β* <*∞*. Let {*X*,*X*_*n*_; *n* ≥ 1} be a sequence of real-valued i.i.d. random variables. We say *X* satisfies the (*α*,*β*)-Chover-type law of the iterated logarithm (and write *X*∈*CTLIL*(*α*,*β*)) if
1.2

From the classical Chover LIL and Definition 1.1, we see that *X*∈*CTLIL*(*α*,1/*α*) (i.e., (1.2) holds with *β*=1/*α*) when *X* has a symmetric stable distribution with exponent *α*∈(0,2).

This paper is devoted to a characterization of *X*∈*CTLIL*(*α*,*β*). The main results are stated in Section 2. We obtain sets of necessary and sufficient conditions for *X*∈*CTLIL*(*α*,*β*) for the five cases: *α* = 2 and 0 < *β* <*∞* (see Theorem 2.1), *α*=2 and *β* = 0 (see Theorem 2.2), 1 < *α* < 2 and −*∞*<*β* <*∞* (see Theorem 2.3), *α* = 1 and −*∞* < *β* < *∞* (see Theorem 2.4), and 0 < *α* < 1 and −*∞* < *β* < *∞* (see Theorem 2.5). The proofs of Theorems 2.1-2.5 are given in Section 4. For proving Theorems 2.1-2.5, three preliminary lemmas are stated in Section 3. Some llustrative examples are provided in Section 5

## 2 Statement of the main results

The main results of this paper are the following five theorems. We begin with the case where *α* = 2 and 0 < *β* <*∞*.

### **Theorem2.1**.

Let 0 < *β* <*∞*. Let {*X*,*X*_*n*_;*n* ≥ 1} be a sequence of i.i.d. real-valued random variables. Then
2.1

if and only if
2.2

For the case where *α* = 2 and *β* = 0, we have the following result.

### **Theorem2.2**.

Let {*X*,*X*_*n*_;*n* ≥ 1} be a sequence of i.i.d. non-degenerate real-valued random variables. Then
2.3

if and only if
2.4

In either case, we have
2.5

### **Remark2.1**

Let *c* be a constant. Note that


Thus, from Theorem 2.2, we conclude that, for any −*∞* <*β* <0, *X* ∉*CTLIL*(2,*β*) for any real-valued random variable *X*.

In the next three theorems, we provide necessary and sufficient conditions for *X* ∈*CTLIL*(*α*,*β*) for the three cases where 1 < *α* < 2 and −*∞* < *β* < *∞*, *α* = 1 and −*∞* <*β* <*∞*, and 0 < *α* <1 and −*∞* <*β* <*∞* respectively.

### **Theorem2.3**.

Let 1<*α*<2 and −*∞* <*β* <*∞*. Let {*X*,*X*_*n*_;*n* ≥ 1} be a sequence of i.i.d. real-valued random variables. Then


if and only if


### **Theorem2.4**.

Let −*∞* <*β* <*∞*. Let {*X*,*X*_*n*_;*n* ≥ 1} be a sequence of i.i.d. real-valued random variables. Then


if and only if


In particular,  and  imply that


### **Theorem2.5**.

Let 0 < *α* <1 and −*∞* <*β* <*∞*. Let {*X*,*X*_*n*_;*n* ≥ 1} be a sequence of i.i.d. real-valued random variables. Then


if and only if


### **Remark2.2**.

From our Theorems 2.1, 2.3, 2.4, and 2.5, a simple and precise characterization of the classical Chover LIL (i.e., *X*∈*CTLIL*(*α*,1/*α*)) is obtained as follows. For 0 < *α*≤ 2, we have


if and only if


Our Theorems 2.1-2.5 also imply the following two interesting results.

### **Corollary2.1**

Let 0 < *α*≤ 2. Let {*X*,*X*_*n*_;*n* ≥ 1} be a sequence of i.i.d. real-valued random variables. Then


if and only if


### **Corollary2.2**.

Let 0 < *α*≤ 2. Let {*X*,*X*_*n*_;*n* ≥ 1} be a sequence of i.i.d. real-valued random variables. Then


if and only if


From our main results Theorems 2.1-2.5 and Corollaries 2.1-2.2 above, an almost unified characterization for 0 < *α*≤ 2 stated as the following result was so kindly presented to us by a referee.

### **Theorem2.6**.

Let 0 < *α*≤ 2. Let {*X*,*X*_*n*_;*n* ≥ 1} be a sequence of i.i.d. real-valued random variables. Assume that  and  whenever . Then


where


## 3 Preliminary lemmas

To prove the main results, we use the following three preliminary lemmas. The first lemma is new and may be of independent interest.

### **Lemma3.1**

Let {*a*_*n*_; *n* ≥ 1} be a sequence of real numbers. Let {*c*_*n*_; *n* ≥ 1} be a sequence of positive real numbers such that
3.1

Then we have

(i) There exists a constant −*∞*<*β*<*∞* such that 3.2

if and only if
3.3

(ii) There exists a constant −*∞*<*β*<*∞* such that


if and only if


(iii) There exists a constant −*∞*<*β*<*∞* such that


if and only if


*Proof* We prove the sufficiency part of Part (i) first. It follows from (3.3) that the set


Note that


We thus conclude that


which ensures (3.2).

We now prove the necessity part of Part (i). For all *b*≠*β*, let *h*=(*b*+*β*)/2. Then


It follows from (3.2) that the set


Note that


We thus have that
3.4

Note that (3.1) implies that


Thus it follows from (3.4) that


i.e., (3.3) holds.

We leave the proofs of Parts (ii) and (iii) to the reader since they are similar to the proof of Part (i). This completes the proof of Lemma 3.1. □

The following result is a special case of Corollary 2 of Einmahl and Li 2005.

### **Lemma3.2**.

Let *b*>0. Let {*X*,*X*_*n*_;*n* ≥ 1} be a sequence of i.i.d. random variables. Then


if and only if


where , *x* ≥ 0.

The following result is a generalization of Kolmogorov-Marcinkiewicz-Zygmund strong law of large numbers and follows easily from Theorems 1 and 2 of Feller 1946.

### **Lemma3.3**.

Let 0 < *α*<2 and −*∞*<*b*<*∞*. Let {*X*,*X*_*n*_;*n* ≥ 1} be a sequence of i.i.d. random variables. Then


if and only if


where  whenever either 1<*α*<2 or *α*=1 and −*∞*<*b*≤ 0.

## 4 Proofs of the main results

In this section, we only give the proofs of Theorems 2.1-2.2. By applying Lemmas 3.1 and 3.3, the proofs of Theorems 2.3-2.5 involves only minor modifications of the proof of Theorem 2.1 and will be omitted.

*Proof of Theorem 2.1* We prove the sufficiency part first. Note that the second part of (2.2) implies that
4.1

We thus see that for all *b*>*β*,


where *h*=(*b*+*β*)/2. Since *β*<*h*<*b*, it follows from (4.1) that


We thus conclude from (2.2) that


which, by applying Lemma 3.2, ensures that
4.2

Since *β*>0, the second part of (2.2) implies that


which ensures that
4.3

Let  and *c*_*n*_=*LLn*, *n* ≥ 1. It then follows from (4.2) and (4.3) that
4.4

By Lemma 3.1, we see that (4.4) is equivalent to


i.e., (2.1) holds.

We now prove the necessity part. By Lemma 3.1, (2.1) is equivalent to (4.4) which ensures that (4.2) holds. By Lemma 3.2, we conclude from (4.2) that
4.5

Since 0 < *β*<*∞*, it follows from (4.5) that


If *β*_1_<*β* then, using the argument in the proof of the sufficiency part, we have that
4.6

Hence, by Lemma 3.1, (4.6) implies that


which is in contradiction to (2.1). Thus (2.2) holds. The proof of Theorem 2.1 is complete. □

*Proof of Theorem 2.2* Using the same argument used in the proof of the sufficiency part of Theorem 2.1, we have from (2.4) that
4.7

Since *X* is a non-degenerate random variable, by the classical Hartman-Wintner-Strassen LIL, we have that


which implies that
4.8

Let  and *c*_*n*_=*LLn*, *n* ≥ 1. It then follows from (4.7) and (4.8) that
4.9

By Lemma 3.1, we see that (4.9) is equivalent to


i.e., (2.5) holds, so does (2.3).

Using the same argument used in the proof of the necessity part of Theorem 2.1, we conclude from (2.3) that


Clearly


Thus (2.4) holds. The proof of Theorem 2.2 is therefore complete. □

## 5 Example

In this section, we provide the following examples to illustrate our main results. By applying Theorems 2.3-2.5, we rededuce the classical Chover LIL in the first example.

### **Example5.1**.

Let 0 < *α*≤ 2. Let *X* be a symmetric real-valued stable random variable with exponent *α*. Clearly,  whenever 1<*α*≤ 2.

For 0 < *α*<2, we have


it follows that


and hence that


Thus, by Theorems 2.3-2.5, *X*∈*CTLIL*(*α*,1/*α*) (i.e., the classical Chover LIL follows).

However, for *α*=2, we have . Hence, by Theorems 2.1 and 2.2, we see that *X*∉*CTLIL*(2,1/2) but *X*∈*CTLIL*(2,0).

From our second example, we will see that *X*∈*CTLIL*(*α*,*β*) for some certain *α* and *β* even if the distribution of *X* is not in the domain of attraction of the stable distribution with exponent *α*.

### **Example5.2**.

Let 0 < *α*≤ 2. Let *d*_*n*_= exp(2^*n*^), *n* ≥ 1. Given −*∞*<*λ*<*∞*. Let *X* be a symmetric i.i.d. real-valued random variable such that


where


Then


Thus the distribution of *X* is not in the domain of attraction of the stable distribution with exponent *α*. Also  whenever either 1<*α*≤ 2 or *α*=1 and *λ*<0. It is easy to see that


and hence that


Thus, by Theorems 2.1-2.5, we have

1. If *α* = 2 and 0 < *λ*<*∞*, then *X*∈*CTLIL*(2,*λ*/2).
2. If *α*=2 and −*∞*<*λ*≤ 0, then *X*∈*CTLIL*(2,0).
3. If 0 < *α*<2, then *X*∈*CTLIL*(*α*,*λ*/*α*).

Our third example shows that *X* may satisfy the other Chover-type LIL studied by Chen and Hu 2012 when *X*∉*CTLIL*(*α*,*β*).

### **Example5.3**.

Define the density function *f*(*x*) of *X* by


where *p*≠0, 0 < *γ*<1, *c*=*c*(*α*,*p*,*γ*) is a positive constant such that . On simplification one can show that for any −*∞*<*b*<*∞*

From Theorem 2.1-2.5, we have

1. If *α* = 2 and *p* < 0, then *X*∈*CTLIL*(2,0).
2. If *α* = 2 and *p* > 0, then *X*∉*CTLIL*(2,*β*) for any 0≤ *β* <*∞*.
3. If 0 < *α* < 2, then *X* ∉*CTLIL*(*α*,*β*) for any −*∞* <*β* <*∞*. However, for 0 < *α* < 2, by Theorem 3.1 in Chen and Hu 2012, we have

where *B*(*x*) is the inverse function of *x*^*α*^/ exp(*p*(log*x*)^*γ*^), *x* ≥*e*.
